# Pikosekundenlaser in der Dermatologie

**DOI:** 10.1007/s00105-023-05144-3

**Published:** 2023-04-26

**Authors:** Lynhda Nguyen, Stefan W. Schneider, Katharina Herberger

**Affiliations:** 1 Laserabteilung, Klinik und Poliklinik für Dermatologie und Venerologie, Universitätsklinikum Hamburg-Eppendorf, Martinistr. 52, 20246 Hamburg, Deutschland; 2 Klinik und Poliklinik für Dermatologie und Venerologie, Universitätsklinikum Hamburg-Eppendorf, Martinistr. 52, 20246 Hamburg, Deutschland

**Keywords:** Laser-induced optical breakdown, Photomeschanisch, Tätowierung, Pigmentierung, Verjüngung, Laser-induced optical breackdown, Photomechanical, Tattoo, Pigmentation, Rejuvenation

## Abstract

**Hintergrund:**

Der Pikosekundenlaser gehört zu den jüngsten in der Dermatologie genutzten Lasersystemen. Ursprünglich wurde er zur Optimierung von Tätowierungsentfernungen entwickelt, Fortschritte in dieser Technologie erweiterten das Indikationsspektrum des Pikosekundenlasers jedoch erheblich.

**Ziel der Arbeit:**

Dieser Artikel gibt eine Übersicht über den technischen Hintergrund sowie die Indikationen des Pikosekundenlasers in der dermatologischen Lasermedizin und erläutert die Möglichkeiten und Grenzen dieses Lasersystems.

**Material und Methoden:**

Grundlage dieses Beitrages sind systematische Literaturanalyse sowie Erfahrungen aus der klinischen Praxis in der universitären Laserambulanz.

**Ergebnisse:**

Der Pikosekundenlaser ermöglicht durch Impulse im Pikosekundenbereich und den Wirkmechanismus des „laser-induced optical breakdown“ eine besonders schonende und effektive Behandlung. Im Vergleich zu den gütegeschalteten Lasern weist der Pikosekundenlaser weniger hitzeinduzierte Nebenwirkungen auf und geht mit einer geringeren Schmerzintensität sowie einer kürzeren Ausfallzeit einher. Insbesondere durch die fraktionierte nichtablative Anwendung haben sich zusätzliche Anwendungsgebiete ergeben, darunter die Hautverjüngung und Narbentherapie.

**Schlussfolgerung:**

Der Pikosekundenlaser findet ein breites Anwendungsspektrum in der dermatologischen Lasermedizin. Die aktuelle Datenlage deutet darauf hin, dass der Laser eine effektive Methode mit einem geringen Nebenwirkungsprofil ist. Um die Wirksamkeit, Verträglichkeit und Patientenzufriedenheit evidenzbasiert beurteilen zu können, sind weitere prospektive Studien notwendig.

## Abstract (en)

**Background:**

The picosecond laser is one of the latest laser systems in dermatology and was originally developed to optimize tattoo removal. Advances in this technology has expanded the use of the picosecond laser to numerous other indications.

**Objectives:**

This article provides an overview of the technical background as well as the indications of the picosecond laser in dermatological laser medicine and elucidates the possibilities and limits of this laser system.

**Materials and methods:**

The article is based on a review of the current literature as well as experience from clinical practice in a university laser department.

**Results:**

The picosecond laser enables a particularly gentle and effective treatment due to ultra-short pulses and the principle of laser-induced optical breakdown. Compared to Q‑switched lasers, the picosecond laser has fewer side effects and is associated with lower pain intensity and shorter downtime. In addition to the removal of tattoos and pigmentary disorders, it is also used in the treatment of scars and rejuvenation.

**Conclusions:**

The picosecond laser has a wide range of indications in dermatological laser medicine. The current data indicate that the laser is an effective method with few side effects. Further prospective studies have to be conducted to assess the efficacy, tolerability and patient satisfaction in an evidence-based manner.

Der Pikosekundenlaser gehört zur neuesten Lasergeneration in der Dermatologie und wurde ursprünglich zur Entfernung unerwünschter Tätowierungen entwickelt. Insbesondere durch die fraktionierte nichtablative Anwendung haben sich in der Praxis zusätzliche Anwendungsgebiete ergeben, darunter die Hautverjüngung und Narbentherapie.

## Technische Grundlagen: das Prinzip der selektiven Gewebewirkung

In den letzten 3 Jahrzehnten hat sich ein breites Spektrum von Lasersystemen im Fachbereich der Dermatologie entwickelt und etabliert. Unterschieden werden langgepulste Laser mit Pulsbreiten im Milli- bis Mikrosekundenbereich und kurzgepulste Laser mit einer Pulsbreite von Nano- bis Pikosekunden. Nach dem physikalischen Wirkprinzip der selektiven Photothermolyse ist für die gezielte Entfernung von Pigmenten eine Pulsdauer erforderlich, die kürzer ist als die thermische Relaxationszeit selbst (engl.: „thermal relaxation time“ [TRT]) [2, 12, 17, 20]. Anders als länger gepulste Lasersysteme, deren Wirkmechanismus überwiegend auf einem thermischen Effekt beruht, basiert die Wirkweise der Pikosekundenlaser auf dem photomechanischen und photoakustischen Effekt. Durch Impulse im Bereich von 10^−9^ bis 10^−12^ s werden Druckwellen induziert, die gezielt Pigmente in Mikropartikel zerteilen, welche anschließend über Phagozytose, Lymphabfluss und transepidermale Elimination entfernt werden [19]. Neben der Behandlung von Pigmentstörungen und Tattoos werden Pikosekundenlaser mittlerweile auch in der Behandlung von Narben und zur Hautverjüngung eingesetzt. Ermöglicht wird dies durch den Wirkmechanismus des „laser-induced optical breakdown“ (LIOB), der mikroskopische Kavitationen in der Dermis verursacht und dadurch die Kollagen- und Elastinsynthese stimuliert [16]. Somit wird eine den fraktioniert ablativen Lasern ähnliche Wirkung erzielt mit dem bedeutenden Unterschied, dass dabei die epidermale Barriere intakt bleibt [15].

Die Pulsdauer im Pikosekundenbereich führt zu einer geringeren Hitzeentwicklung, welche das Risiko für kollaterale, thermische Schäden verringert. Durch hohe thermische Energie werden unerwünschte Nebenwirkungen wie Verbrennungen sowie postinflammatorische Hyper- und Hypopigmentierungen verursacht. Somit sind Pikosekundenlaser – insbesondere bei einer Wellenlänge von 1064 nm – für Patienten mit dunklerem Hauttyp geeigneter als gütegeschaltete Lasersysteme [9, 14, 32, 40]. Darüber hinaus ermöglicht die Wirkweise der Pikosekundenlaser eine verminderte Schmerzintensität und eine kurze Ausfallzeit [29].

## Indikationen

### Tätowierungen

Eine im Jahr 2016 veröffentlichte Studie berichtete, dass die Prävalenz für Tätowierungen in der deutschen Bevölkerung bei 37 % liegt und eine steigende Tendenz aufweist [6]. Mittlerweile sind Tattoos in allen Altersgruppen und sozialen Schichten weit verbreitet. Mit der größeren Nachfrage nach Tätowierungen steigt jedoch auch der Bedarf an effektiven Methoden zur Tätowierungsentfernung [3, 22, 39]. Lange Zeit waren die gütegeschalteten Nanosekundenlaser hierbei das Mittel der Wahl [33]. Da die thermische Relaxationszeit (TRT) der Farbpigmente jedoch bei ≤ 10 ns liegt, sind sie theoretisch ein ideales Ziel für einen kürzeren Puls im Bereich von Pikosekunden [34].

Vier prospektive Vergleichsstudien analysierten die Wirksamkeit und Verträglichkeit der Pikosekundenlaser und der Nanosekundenlaser an schwarzen und blauen Tätowierungen [18, 23, 29, 35]. Sie weisen darauf hin, dass Behandlungen mit 532-nm/1064-nm-Pikosekundenlasern bessere klinische Ergebnisse mit einer geringeren Nebenwirkungsrate erzielen können.

**Figure 1:**
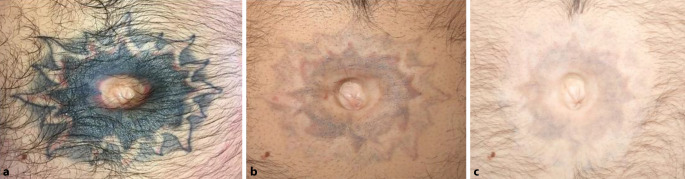

Die Farbe der Tätowierung ist ein wichtiger Prädiktor für das Ergebnis der Behandlung. Besonders die Behandlung gelber bis oranger Farbpigmente erwies sich in der Vergangenheit als schwierig. Alabdulrazzaq et al. behandelten gelbe Farbpigmente mit dem 532-nm-Pikosekundenlaser und berichteten über eine Entfernung von 75 % nach 2 bis 4 Sitzungen [1]. Der 532/1064-nm-Pikosekundenlaser scheint in der Entfernung vielfarbiger Tätowierungen suffiziente klinische Ergebnisse zu erreichen [23]. Die Abb. 1 zeigt eine umfassende Aufhellung eines bunten Tattoos. Im Abstand von 4 Wochen erfolgten 9 Sitzungen mit einem 1064-nm-Pikosekundenlaser (PicoPlus®, Lutronic Co. Ltd., Hamburg, Deutschland) mit folgenden Einstellungen: 3–8 mm Spotgröße, 0,6–1,9 J/cm^2^ Fluenz, 2 Hz. Rote Pigmente wurden mit einer Wellenlänge von 532 nm (3–5,3 mm Spotgröße, 0,4–0,85 J/cm^2^ Fluenz, 2 Hz) entfernt. Zur Minderung von Schmerzen erfolgte der Einsatz eines Kaltluftgerätes (Cryo 6®, Zimmer Aesthetics, Neu-Ulm, Deutschland). In der Nachkontrolle konnten keine Narben oder Blasen sowie wenige Krusten dokumentiert werden. Jüngere Erfahrungen zeigen jedoch, dass ein längeres Behandlungsintervall von mindestens 6 Wochen zu einer Verringerung der benötigten Sitzungen führen kann, da der Abtransport von Pigmenten nach 4 Wochen offenbar noch nicht abgeschlossen ist [1, 21]. Zur Ermittlung des idealen Behandlungsintervalls sind weitere klinische Studien notwendig.

### Epidermale und dermale Hyperpigmentierung

#### Melasma

**Figure 2:**
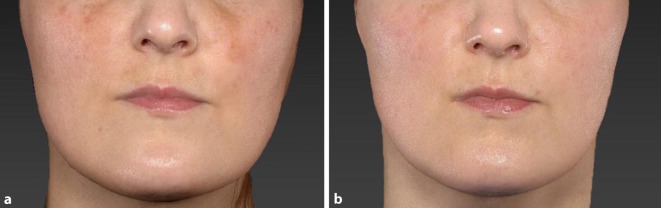

Die Behandlung des Melasmas stellt unter anderem aufgrund der hohen Rezidivrate eine therapeutische Herausforderung dar. Aktuell ist die Therapie der Wahl die depigmentierende Triple-Kombinationsbehandlung bestehend aus Hydrochinon, Retinoid und Steroid [30]. Eine Studie im Seitenvergleich beobachtete eine schnellere Aufhellung der Makulae unter einer Therapie mit einem Alexandrit-Pikosekundenlaser als mit einem Alexandrit-Nanosekundenlaser [28]. Eine therapeutische Überlegenheit des Alexandrit-Pikosekundenlasers zur Triple-Therapie ließ sich nicht nachweisen [41]. Daten der letzten Jahre deuten darauf hin, dass der 1927-nm-Thulium-Laser eine effektive und sichere Behandlungsmethode ist [24, 27]. Insgesamt führten diese Interventionen zwar zur deutlichen Besserung. Jedoch trat in allen Behandlungsgruppen eine hohe Rückfallrate auf [31]. IPL(„intense pulsed light“)-Geräte sowie gütegeschaltete Nd:YAG(Neodym-dotierter Yttrium-Aluminium-Granat)-Laser werden aufgrund des hohen Risikos einer postinflammatorischen Hyperpigmentierung nicht empfohlen. Im vorliegenden Fall (Abb. 2) wurde eine Patientin mit Melasma neben dem kollimierten Handstück des 1064-nm-Pikoskundenlasers (8 mm Spotgröße, 0,7–1,0 J/cm^2^ Fluenz, 10 Hz) zusätzlich mit dem fraktionierten Handstück des 1064-nm-Pikosekundenlasers (10 mm Spotgröße, 0,22–0,3 J/cm^2^, 10 Hz) behandelt. Dabei wurde der Strahlenfokus mithilfe des justierbaren Handstücks in den 3 Tiefen (Tiefe 1–3) eingestellt. Insgesamt fanden 4 Sitzungen im Abstand von 8 Wochen statt. Das Kaltluftgerät reduzierte suffizient Schmerzen. Eine vorübergehende Rötung von etwa 30 min wurde beobachtet. Ob der Einsatz der Pikosekundentechnologie jedoch einen Vorteil gegenüber den bereits beschriebenen Methoden aufweist, ist derzeit nicht gesichert. Es ist mit einer ähnlichen Rückfallrate wie bei anderen Systemen zu rechnen, diese sollte Teil des Aufklärungsgesprächs sein.

#### Café-au-lait-Flecken

**Figure 3:**
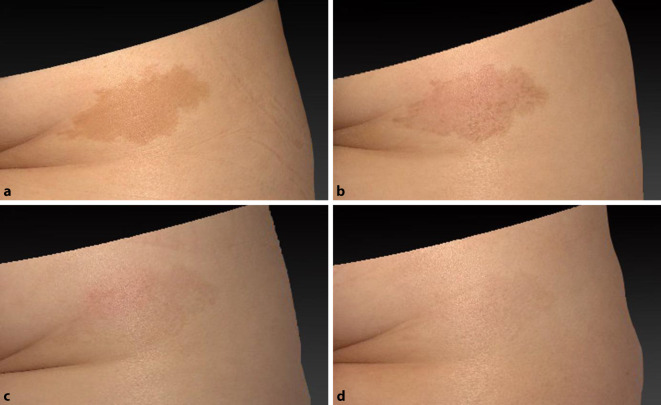

Die Morphologie von Café-au-lait-Flecken scheint mit dem Ansprechverhalten auf Lasertherapien zu korrelieren. So führt eine Lasertherapie an irregulär geformten Café-au-lait-Flecken zu guten bis exzellenten Ergebnissen, während Makulae mit weichen Kanten mehr Sitzungen benötigen und ein erhöhtes Risiko für Hypopigmentierungen aufweisen [5]; 755-nm-Pikosekundenlaser sowie 755-nm- und 532-nm-Nanosekundenlaser sind bei Café-au-lait-Flecken im gleichen Maße effektiv. Der Pikosekundenlaser weist weniger Nebenwirkungen auf [8]. Die Abb. 3 veranschaulicht den Therapieverlauf eines irregulär geformten Café-au-lait-Flecks. Eine probatorische Behandlung mit dem 660-nm-ruby-like variant YAG-Laser-Pikosekundenlaser (3 mm Spotgröße, 0,6 J/cm^2^ Fluenz, 5 Hz) führte zu Hyperpigmentierungen (Abb. 3b). Es folgte der Einsatz des kollimierten Handstückes eines 532-nm-Pikosekundenlasers (3–5,3 mm Spotgröße, 0,1–0,5 J/cm^2^ Fluenz, 2–5 Hz). Während der dritten und vierten Behandlung wurde zudem das fraktionierte Handstück eines 532-nm-Pikosekundenlasers (5,3 mm Spotgröße, 0,04 J/cm^2^, 10 Hz) eingesetzt. Zur Schmerzlinderung wurde ein Kaltluftgerät eingesetzt. Nach 5 Behandlungen konnte eine sehr gute bis exzellente Aufhellung dokumentiert werden.

#### Nävus Ota

Die gütegeschalteten Rubin- und Nd:YAG-Laser stellten viele Jahre das Mittel der ersten Wahl in der Therapie dieser pigmentierten Fehlbildung dar. Allerdings war die Therapie mit zum Teil erheblichen Schmerzen und Nachwirkungen wie Krusten und Dyspigmentierungen verbunden. Frühere Therapieansätze mittels Dermabrasion, Hauttransplantationen oder abtragenden Lasersystemen führten zu keinem zufriedenstellenden kosmetischen Ergebnis. In einer randomisierten, kontrollierten Studie erzielte der Alexandrit-Pikosekundenlaser eine exzellente bis komplette Aufhellung des Nävus Ota bei gleichzeitig geringerer Anzahl an notwendigen Sitzungen im Vergleich zum Alexandrit-Nanosekundenlaser. Weiterhin war die Schmerzintensität signifikant gemindert [13]. Da der Nd:YAG-Laser eine höhere Eindringtiefe als der Alexandrit-Laser hat und er die dermalen Pigmente unter Schonung der epidermal gelegenen Pigmentierung selektiver fokussiert, ist eine schonendere Behandlung durch den Nd:YAG-Pikosekundenlaser zu erwarten. Dies spiegelt unsere Erfahrung insbesondere bei dunkleren Hauttypen mit dem kollimierten Handstück des 1064-nm-Pikosekundenlasers wider (5 bis 10 Behandlungen im Abstand von 2 bis 4 Wochen mit folgenden Parametern: 7–8 mm Spotgröße, 1,0–1,4 J/cm^2^ Fluenz, 5–10 Hz). Die Behandlung ist ohne Betäubung möglich, eine Krustenbildung ist nicht zu beobachten.

### Aknenarben

Vernarbungen nach Acne vulgaris sind weit verbreitet und können den Betroffenen psychosozial schwer belasten. Neben topischen Therapien und Microneedling haben sich verschiedene Lasersysteme in der Behandlung von Aknenarben etabliert. Brauer et al. beschreiben eine Volumenzunahme der atrophen Aknenarben um rund 24 % nach 6 Alexandrit-Pikosekundenlaser-Behandlungen. Äquivalent zum klinischen Ergebnis zeigten histologische Untersuchungen eine zunehmende Dichte an Elastin- und Kollagenfasern [7]. Bisherige Analysen zeigen dabei keinen deutlichen Unterschied zwischen dem fraktionierten Nd:YAG-Pikosekundenlaser und dem fraktionierten CO_2_-Laser [37]. Der Pikosekundenlaser birgt insbesondere bei dunklen Hauttypen eine geringere Gefahr für postinflammatorische Dyspigmentierungen [37]. Um relevante Behandlungseffekte zu ermöglichen, sind allerdings mindestens 4 bis 6 Sitzungen in einem Abstand von 2 bis 4 Wochen erforderlich. Sirithanabadeekul et al. behandelten 25 Patienten mit dem Pikosekundenlaser und CO_2_-Laser im Split-Side-Design und wiesen eine gleichwertige Effektivität mit höherem Sicherheitsprofil des Pikosekundenlasers nach [36]. Lee et al. haben einen 755-nm-Pikosekundenlaser mit einem ablativen CO_2_-Laser bei atrophen Aknenarben verglichen und deutlich bessere klinische Ergebnisse mit dem Pikosekundenlaser beobachtet [26]. Wenige weitere Studien verglichen den Pikosekundenlaser mit dem nichtablativen 1550-nm-Erbium:Glass-Laser und stellten eine gleichwertige bis bessere Wirkung mit hohem Sicherheitsprofil fest [10, 25]. Weitere prospektive Studien sind notwendig, um die Effektivität und Sicherheit mit etablieren Verfahren wie dem Erbium:YAG- und CO_2_-Laser ausreichend beurteilen zu können.

### Hautverjüngung

**Figure 4:**
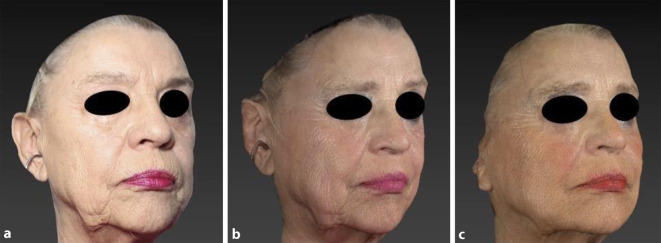

Verschiedene Studien weisen auf eine wirksame und sichere Behandlung von lichtbedingt gealterter Haut mit Pikosekundenlasern hin. Weiss et al. behandelten mit einem Alexandrit-Pikosekundenlaser Falten der perioralen und periokulären Region. Serielle Biopsien nach 1, 3 und 6 Monaten wiesen dichter arrangierte Kollagen- und Elastinfasern nach [42]. Dabei wird angenommen, dass der Wirkmechanismus auf dem von Pikosekundenlasern induzierten LIOB beruht. Durch Entstehung mikroskopischer Kavitationen in der Dermis wird ein Remodeling der Kollagen- und Elastinfasern hervorgerufen [4, 11, 38]. Die ausgeprägte Dermatochalasis einer 83-jährigen Patientin ließ sich in 6 Sitzungen im Abstand von 2 bis 4 Wochen zufriedenstellend behandeln (Abb. 4). Nach einer topischen Anästhesie wurde das kollimierte Handstück eines 1064 nm-Pikosekundenlasers (9–10 mm Spotgröße, 0,25–0,5 J/cm^2^ Fluenz, 10 Hz) eingesetzt. Ab der dritten Sitzung konnte auf eine Lokalanästhesie verzichtet werden. Kombiniert mit Augmentationen der Jochbögen und der Mentolabialfalten mittels hochvernetzter Hyaluronsäure (Restylane Defyne 3 ml, Fa. Galderma) wurden eine deutliche Hautstraffung, besonders entlang der Kinn-Kiefer-Linie, und eine Verfeinerung der Hauttextur erzielt. Dieses Vorgehen wurde gewählt, da aufgrund der Einnahme des Antikoagulans Apixaban keine ablative Resurfacing-Methode möglich war.

**Figure 5:**
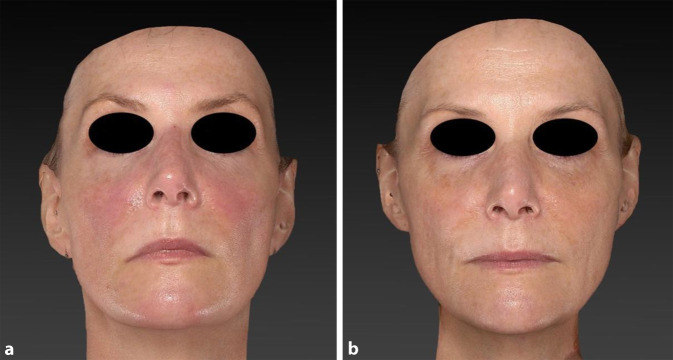

Im folgenden Behandlungsbeispiel konnte durch Reduktion von Lentigines und Hautrötungen ebenfalls ein jüngeres Aussehen wiederhergestellt werden (Abb. 5). Hierzu wurde in 4 Sitzungen im Abstand von 4 Wochen ein 1064-nm-Pikosekundenlaser (kollimiertes Handstück: 6–8 mm Spotgröße, 0,7–1,0 J/cm^2^ Fluenz, 10 Hz; fraktioniertes Handstück: 8 mm Pulsbreite, 0,26 J/cm^2^ Fluenz, 10 Hz) über die gesamte Gesichtsfläche eingesetzt. Zusätzlich erfolgte die Behandlung größerer Lentigines mit einem 660-nm-Pikosekundenlaser (3 mm Spotgröße, 0,9 J/cm^2^ Fluenz, 2 Hz). Inwieweit die Laserbehandlung Einfluss auf den Rückgang der Rötung hat, ist in diesem Fall nicht sicher zu erklären.

Zusammenfassend findet der Pikosekundenlaser in der dermatologischen Praxis ein breites Anwendungsspektrum. Die Tab. 1 fasst die wichtigsten Indikationen des Pikosekundenlasers zusammen und gibt die Evidenz und alternative Therapiemöglichkeiten für die jeweiligen Einsatzgebiete wieder.

**Table 1:** 

Indikationen	Evidenz Pikosekundenlaser	Besonderheiten/therapeutische Einschätzung Pikosekundenlaser	Therapiealternativen
Tätowierung	+++	Geringere Nebenwirkungen Weniger Sitzungen, größeres Behandlungsintervall	Gütegeschaltete Laser +++ Dermabrasion +++
Melasma	+	4 bis 6 Sitzungen nötig Aufklärung über Rekurrenz im Sommer Bei zu hohen Energien Verschlechterung möglich, v. a. bei Hauttyp III–IV	Depigmentierende Triple-Kombinationsbehandlung +++ 1927-nm-Thulium-Laser + Gütegeschaltete Laser ++ IPL ++ Chemisches Peeling ++
Café-au-lait-Fleck	++	Gleichmäßigere Aufhellung durch Einsatz fraktionierter Handstücke (532 nm) Wie bei anderen Systemen Rekurrenz möglich	Gütegeschaltete Laser +++ Dermabrasion ++ Kryotherapie ++
Nävus Ota	++	Geringe Schmerzen Schnellere Aufhellung durch kürzeres Behandlungsintervall (2 bis 3 Wochen) Kurze Ausfallzeit (keine Krusten, Bläschen)	Gütegeschaltete Laser +++ Dermabrasion ++ Kryotherapie ++
Aknenarben	+	Behandlung dunkler Hauttypen möglich Mindestens 4 bis 6 Sitzungen nötig Kurze Ausfallzeit (24 h Rötungen, keine Krusten) Schüsselförmige Narben schwierig zu behandeln, ggf. Multistacking	Chemische Peelings ++ Fraktionierter CO_2_-Laser +++ Microneedling +++ Dermabrasion ++ Operative Narbentherapie +++
Rejuvenation	++	Behandlung dunkler Hauttypen möglich Mindestens 3 bis 4 Sitzungen nötig Kurze Ausfallzeit (24 h Rötungen, keine Krusten) Minderung feiner Fältchen, bei fortgeschrittenen Falten keine ausreichende Wirkung	Fraktionierter CO_2_-Laser +++ Radiofrequenz-Microneedling ++ Augmentation +++ Chirurgische Gesichtsstraffung +++

Evidenz: − keine, + mäßig, ++ gut, +++ sehr gut

## Fazit für die Praxis

- Der Pikosekundenlaser hat im Fachbereich der Dermatologie ein breites Einsatzspektrum und findet u. a. Anwendung in der Entfernung von Tätowierungen, Behandlung dermaler und epidermaler Pigmentstörungen und Induktion des Gewebeumbaus.
- Die Impulsdauer im Pikosekundenbereich erlaubt eine gezielte Fragmentierung von Pigmenten und eine thermische Schonung des umgebenen Gewebes.
- Nach aktueller Datenlage ist der Pikosekundenlaser für Patienten mit den Hauttypen IV–VI nach Fitzpatrick geeigneter als gütegeschaltete Laser, da er ein vermindertes Risiko für Hypo- und Hyperpigmentierungen hat.
- Sofortige Reaktionen unter der Laserbehandlung wie Erythem, Ödeme und Schmerzen scheinen milder als bei gütegeschalteten Lasersystemen zu verlaufen und klingen in der Regel innerhalb weniger Stunden bis Tage ab.
- Aufgrund der derzeit hohen Anschaffungskosten eines Pikosekundenlasers ist dessen Einsatz zunächst spezialisierten Zentren vorbehalten.
- Es bedarf weiterer Studien, um das Potenzial des Pikosekundenlasers auf lange Sicht beurteilen zu können.

## References

[1] Alabdulrazzaq H, Brauer JA, Bae YS (2015). Clearance of yellow tattoo ink with a novel 532-nm picosecond laser. Lasers Surg Med.

[2] Anderson RR, Parrish JA (1983). Selective photothermolysis: precise microsurgery by selective absorption of pulsed radiation. Science.

[3] Armstrong ML, Stuppy DJ, Gabriel DC (1996). Motivation for tattoo removal. Arch Dermatol.

[4] Balu M, Lentsch G, Korta DZ (2017). In vivo multiphoton-microscopy of picosecond-laser-induced optical breakdown in human skin. Lasers Surg Med.

[5] Belkin DA, Neckman JP, Jeon H (2017). Response to laser treatment of cafe au lait macules based on morphologic features. JAMA Dermatol.

[6] Borkenhagen A, Mirastschijski U, Petrowski K (2019). Tattoos in Germany: prevalence, demographics, and health orientation. Bundesgesundheitsblatt Gesundheitsforschung Gesundheitsschutz.

[7] Brauer JA, Kazlouskaya V, Alabdulrazzaq H (2015). Use of a picosecond pulse duration laser with specialized optic for treatment of facial acne scarring. JAMA Dermatol.

[8] Cen Q, Gu Y, Luo L (2021). Comparative effectiveness of 755-nm picosecond laser, 755- and 532-nm nanosecond lasers for treatment of cafe-au-lait macules (CALms): a randomized, split-lesion clinical trial. Lasers Surg Med.

[9] Chan MWM, Shek SY, Yeung CK (2019). A prospective study in the treatment of lentigines in asian skin using 532 nm picosecond Nd:YAG laser. Lasers Surg Med.

[10] Chayavichitsilp P, Limtong P, Triyangkulsri K (2020). Comparison of fractional neodymium-doped yttrium aluminum garnet (Nd:YAG) 1064-nm picosecond laser and fractional 1550-nm erbium fiber laser in facial acne scar treatment. Lasers Med Sci.

[11] Dierickx C (2018). Using normal and high pulse coverage with picosecond laser treatment of wrinkles and acne scarring: long term clinical observations. Lasers Surg Med.

[12] Freedman JR, Kaufman J, Metelitsa AI (2014). Picosecond lasers: the next generation of short-pulsed lasers. Semin Cutan Med Surg.

[13] Ge Y, Yang Y, Guo L (2020). Comparison of a picosecond alexandrite laser versus a Q-switched alexandrite laser for the treatment of nevus of Ota: A randomized, split-lesion, controlled trial. J Am Acad Dermatol.

[14] Guss L, Goldman MP, Wu DC (2017). Picosecond 532 nm neodymium-doped yttrium ayluminium garnet laser for the treatment of solar lentigines in darker skin types: safety and efficacy. Dermatol Surg.

[15] Habbema L, Verhagen R, Van Hal R (2013). Efficacy of minimally invasive nonthermal laser-induced optical breakdown technology for skin rejuvenation. Lasers Med Sci.

[16] Habbema L, Verhagen R, Van Hal R (2012). Minimally invasive non-thermal laser technology using laser-induced optical breakdown for skin rejuvenation. J Biophotonics.

[17] Henley JK, Zurfley F, Ramsey ML (2022). Laser Tattoo Removal.

[18] Herd RM, Alora MB, Smoller B (1999). A clinical and histologic prospective controlled comparative study of the picosecond titanium:sapphire (795 nm) laser versus the Q-switched alexandrite (752 nm) laser for removing tattoo pigment. J Am Acad Dermatol.

[19] Ho DD, London R, Zimmerman GB (2002). Laser-tattoo removal—a study of the mechanism and the optimal treatment strategy via computer simulations. Lasers Surg Med.

[20] Kasai K (2017). Picosecond laser treatment for tattoos and benign cutaneous pigmented lesions (secondary publication). Laser Ther.

[21] Kauvar ANB, Keaney TC, Alster T (2017). Laser treatment of professional tattoos with a 1064/532-nm dual-wavelength picosecond laser. Dermatol Surg.

[22] Kent KM, Graber EM (2012). Laser tattoo removal: a review. Dermatol Surg.

[23] Kono T, Chan HHL, Groff WF (2020). Prospective comparison study of 532/1064 nm picosecond laser vs 532/1064 nm nanosecond laser in the treatment of professional tattoos in Asians. Laser Ther.

[24] Kurmus G, Tatliparmak A, Aksoy B (2019). Efficacy and safety of 1927 nm fractional Thulium fiber laser for the treatment of melasma: a retrospective study of 100 patients. J Cosmet Laser Ther.

[25] Kwon HH, Yang SH, Cho YJ (2020). Comparison of a 1064-nm neodymium-doped yttrium aluminum garnet picosecond laser using a diffractive optical element vs. a nonablative 1550-nm erbium-glass laser for the treatment of facial acne scarring in Asian patients: a 17-week prospective, randomized, split-face, controlled trial. J Eur Acad Dermatol Venereol.

[26] Lee DW, Ryu H, Choi HJ (2022). Improvement in linear depressed atrophic scar using 755-nm picosecond alexandrite laser vs. ablative fractional carbon dioxide laser. J Cosmet Laser Ther.

[27] Lee HM, Haw S, Kim JK (2013). Split-face study using a 1,927-nm thulium fiber fractional laser to treat photoaging and melasma in Asian skin. Dermatol Surg.

[28] Lee MC, Lin YF, Hu S (2018). A split-face study: comparison of picosecond alexandrite laser and Q-switched Nd:YAG laser in the treatment of melasma in Asians. Lasers Med Sci.

[29] Lorgeou A, Perrillat Y, Gral N (2018). Comparison of two picosecond lasers to a nanosecond laser for treating tattoos: a prospective randomized study on 49 patients. J Eur Acad Dermatol Venereol.

[30] Mckesey J, Tovar-Garza A, Pandya AG (2020). Melasma Treatment: An Evidence-Based Review. Am J Clin Dermatol.

[31] Neagu N, Conforti C, Agozzino M et al (2021) Melasma treatment: a systematic review. J Dermatolog Treat: 1–39. 10.1080/09546634.2021.1914313 10.1080/09546634.2021.1914313 33849384

[32] Negishi K, Akita H, Matsunaga Y (2018). Prospective study of removing solar lentigines in Asians using a novel dual-wavelength and dual-pulse width picosecond laser. Lasers Surg Med.

[33] Passeron T, Genedy R, Salah L (2019). Laser treatment of hyperpigmented lesions: position statement of the European Society of Laser in Dermatology. J Eur Acad Dermatol Venereol.

[34] Raulin C, Schonermark MP, Greve B (1998). Q-switched ruby laser treatment of tattoos and benign pigmented skin lesions: a critical review. Ann Plast Surg.

[35] Ross V, Naseef G, Lin G (1998). Comparison of responses of tattoos to picosecond and nanosecond Q-switched neodymium: YAG lasers. Arch Dermatol.

[36] Sirithanabadeekul P, Tantrapornpong P, Rattakul B (2021). Comparison of fractional picosecond 1064-nm laser and fractional carbon dioxide laser for treating atrophic acne scars: a randomized split-face trial. Dermatol Surg.

[37] Sirithanabadeekul P, Tantrapornpong P, Rattakul B et al (2020) Comparison of fractional picosecond 1064-nm laser and fractional carbon dioxide laser for treating atrophic acne scars: a randomized split-face trial. Dermatol Surg. 10.1097/DSS.0000000000002572 10.1097/DSS.0000000000002572 32910030

[38] Tanghetti EA, Hoffmann KA, Hoffmann K (2017). Short-pulsed laser for the treatment of tattoos, pigmented lesions, scars and rejuvenation. Semin Cutan Med Surg.

[39] Varma S, Lanigan SW (1999). Reasons for requesting laser removal of unwanted tattoos. Br J Dermatol.

[40] Wang CC, Sue YM, Yang CH (2006). A comparison of Q-switched alexandrite laser and intense pulsed light for the treatment of freckles and lentigines in Asian persons: a randomized, physician-blinded, split-face comparative trial. J Am Acad Dermatol.

[41] Wang YJ, Lin ET, Chen YT (2020). Prospective randomized controlled trial comparing treatment efficacy and tolerance of picosecond alexandrite laser with a diffractive lens array and triple combination cream in female asian patients with melasma. J Eur Acad Dermatol Venereol.

[42] Weiss RA, Mcdaniel DH, Weiss MA (2017). Safety and efficacy of a novel diffractive lens array using a picosecond 755 nm alexandrite laser for treatment of wrinkles. Lasers Surg Med.

